# Ultrastructural insights into early myoblast differentiation induced by shockwave stimulation

**DOI:** 10.3389/fphys.2025.1636931

**Published:** 2025-07-23

**Authors:** Larisa Ryskalin, Federica Fulceri, Paola Soldani, Maria Cristina D’Agostino, Gabriele Morucci, Stefania Moscato, Marco Gesi

**Affiliations:** ^1^Department of Translational Research and New Technologies in Medicine and Surgery, University of Pisa, Pisa, Italy; ^2^Shockwave Unit, Rehabilitation Department, IRCCS Humanitas Research Hospital, Rozzano, Milano, Italy; ^3^Department of Clinical and Experimental Medicine, University of Pisa, Pisa, Italy

**Keywords:** shockwave therapy, myoblasts, muscle injury, myogenesis, transmission electron microscopy, protein compartmentalization, myogenic differentiation markers

## Abstract

**Introduction:**

Extracorporeal shockwave therapy (ESWT) is a non-invasive therapeutic modality that uses high-energy acoustic waves (shockwaves, SW) to restore local homeostasis and stimulate tissue healing and regeneration through mechanotransduction. ESWT has gained popularity in treating numerous musculoskeletal indications such as tendinopathies, plantar fasciitis, bony non-unions, and stress fractures, with proven benefits in reducing pain, enhancing recovery, and in some cases preventing recurrence. In contrast, SW application in muscle injuries remains less investigated. Some clinical studies have shown promising results of ESWT for treating muscle injuries. Preclinical animal studies suggest that SW can improve muscle microcirculation, reduce inflammation, and accelerate tissue regeneration. *In vitro* studies, however, reported conflicting data regarding the effects of SW on muscle cells, with little data on ultrastructural changes supporting clinical results.

**Methods:**

This study aimed to evaluate the ultrastructural effects of SW on C2C12 myoblasts. We applied 500 pulses with an Energy Flux Density of 0.1 mJ/mm^2^, 4 Hz, at a distance of 5 cm between the SW applicator and cell culture in a 37°C water bath. Evaluations were conducted at 24 h, 72 h, and up to 7 days post-treatment, including cell viability, Western blot, histomorphometry, and ultrastructural analysis. Immunocytochemistry for Myoblast Determination Protein 1 (MyoD) and Myogenin (MyoG) was performed to characterize subcellular distribution.

**Results:**

Light and electron microscopy revealed that SW stimulation induced significant morphological changes, including increased cell elongation and ultrastructural features suggesting early fusion events. These changes correlated with a rise in the percentage of multinucleated cells, indicative of early myoblast differentiation. Despite this, Western blot analysis showed no significant differences in total MyoD and MyoG levels. However, immunogold electron microscopy demonstrated a marked increase in nuclear localization of both markers in treated cells, aligning with their roles in myogenic differentiation.

**Discussion:**

These findings suggest SW promotes early myogenic progression through enhanced nuclear translocation of key regulatory proteins, rather than altering expression levels. Exploring SW-induced ultrastructural changes may offer new perspectives on early steps of myogenesis and holds promise for disclosing novel hypotheses on SW biological underpinning and expanding translational ESWT application in muscle injuries and sports medicine.

## 1 Introduction

Extracorporeal shockwave therapy (ESWT) is a non-invasive treatment that works by the emission of high-amplitude biphasic pulsed acoustic waves (shockwaves, SW) that rapidly cycle between positive and negative pressures onto the area being treated (Huang et al., 2013; Cheng and Wang, 2015; Simplicio et al., 2020). Thus, acting as a mechanical stimulus, SW can exert its beneficial effects and promote healing processes via mechanotransduction, a process by which cells convert mechanical stimuli into biochemical signals, thereby promoting tissue regeneration and healing (Frairia and Berta, 2012; Notarnicola and Moretti, 2012; Agostino et al., 2015), mainly by modulating inflammation (Sukubo et al., 2015) as well as stimulating angiogenesis (Goertz et al., 2014; Tepeköylü et al., 2018; Wang et al., 2021) and stem cell activities (Tepeköylü, et al., 2013).

Over the last decade, ESWT has become a popular and safe treatment option for various musculoskeletal conditions, ranging from tendinopathies to bone healing disturbances (d’Agostino et al., 2015; Burton, 2021; Kwok et al., 2022; Wuerfel et al., 2022; Lv et al., 2023; Pellegrino et al., 2023; Fulceri et al., 2024a; Majidi et al., 2024). From a clinical perspective, ESWT has proven to b effective also in relieving pain, promoting functional recovery, and reducing disease recurrence in bone and soft-tissue disorders (Ramon et al., 2020; Xu et al., 2019; Liu et al., 2023; Ryskalin et al., 2023; Fulceri et al., 2024b; Xue et al., 2024; Santilli et al., 2025).

On the other hand, its use for acute muscle injuries and chronic muscle conditions is of more recent development (Astur et al., 2015; Ramon et al., 2015; Fleckenstein et al., 2016; Liu et al., 2023; Mazin et al., 2023).

In sports medicine, ESWT has demonstrated benefits in treating injured muscle tissue and painful muscle hematomas and has been associated with reduced layoff times and lower re-injury rates among athletes (Astur et al., 2015; Mazin et al., 2023).

At the same time, preclinical research on the effects of ESWT on muscle tissue has also rapidly expanded, yielding promising results (Kisch et al., 2016; Zissler et al., 2017; Langendorf et al., 2020; Wuerfel et al., 2022; Wang et al., 2024). *In vivo* studies on animal models suggest that ESWT can improve muscle microcirculation, stimulate tissue regeneration, and accelerate repair processes by affecting humoral and cellular factors (Lee and Cho, 2013; Kisch et al., 2016; Zissler et al., 2017; Huang et al., 2021; Lee et al., 2023; Wang et al., 2024). Moreover, Radial Pressure Waves (RPW) reduced inflammatory response and enhanced muscle regeneration in rabbits with acute biceps femoris injury (Wang et al., 2024). In rabbits, RPW could also alleviate immobilization-induced contracture and reduce the molecular manifestations of muscle fibrosis (Huang et al., 2021). Another study found that capillary blood flow is immediately enhanced after a single session of high-energy SW in the muscles of rats, with even greater effects after repeated sessions (Kisch et al., 2016).

On the contrary, little is known about the biological responses elicited by SW when applied to an *in vitro* model of skeletal muscle tissue. So far, only a few studies have investigated the rationale for applying SW to muscle tissue using *in vitro* experimental setups (Hansen et al., 2017; Mattyasovszky et al., 2018; Wang et al., 2017). Therefore, the exact mechanisms underlying ESWT-induced beneficial effects, such as myogenesis, still remain largely unexplored.

Skeletal muscle differentiation, myogenesis, is a finely tuned multistep process in which single mononuclear and elongated cells (i.e., myoblasts) arrest cell division and then start to elongate and fuse, forming multinucleated syncytia delimited by a single plasma membrane called myotubes (Weintraub, 1993; Buckingham et al., 2003). This, in turn, is essential for muscle growth and maintenance and for repairing injured muscles (Kook et al., 2008). This complex sequence of events, which occurs both in muscle development and regeneration, is governed by the coordinated expression of muscle-specific transcription factors belonging to the Myogenic Regulatory Factor (MRF) family, such as the Myoblast Determination Protein 1 (MyoD) and Myogenin (MyoG) (Sabourin and Rudnicki, 2000; Hernández-Hernández et al., 2017). These latter are exclusively expressed in cells committed to the myogenic lineage and become active in a time-correlated manner. MyoD is essential for myoblast identity and acts early in myogenesis to determine myogenic fate, whereas MyoG acts at a later stage, and it is essential to drive the decisive events of myogenic differentiation, such as myoblast fusion (Hasty et al., 1993; Perry and Rudnick, 2000; Zhang et al., 2020).

As far as we know, the current understanding of SW-induced effects mainly relies on gene expression analyses and biochemical data, with limited literature on the ultrastructural changes related to its positive clinical results.

Therefore, the present study aims to investigate for the first time whether SW can promote ultrastructural changes indicative of myogenesis. For this purpose, a primary line of murine myoblasts, namely, the C2C12 cell line, was used, which represents a cell line derived from mouse satellite cells that faithfully mimics skeletal muscle differentiation (Tang et al., 2018); thus, it represents an excellent and well-established model to study myogenic regulation and its responses to stimulation (Luo et al., 2019). In particular, the myogenic response to SW stimulation was studied by evaluating the morpho-functional and ultrastructural changes in fusion-competent myoblast cells towards the formation of multinucleate syncytia as well as the time course expression pattern of the myogenic markers, MyoD and MyoG.

Through these experiments, we seek to provide novel morphological/ultrastructural evidence of the potential of SW in promoting myoblast transition into a multinucleated cell, syncytium. This, in turn, holds the significant therapeutic promise of ESWT for promoting muscle healing and regeneration.

## 2 Materials and methods

### 2.1 Cell culture

All experiments were performed using murine C2C12 myoblasts purchased from the Istituto Zooprofilattico Sperimentale of Lombardia and Emilia Romagna (Brescia, Italy). The cells were maintained in DMEM growth medium (Euroclone, Pero, MI, Italy) supplemented with 10% Foetal Bovine Serum (Euroclone) and 1% penicillin at 37°C in a controlled atmosphere containing 5% CO_2_ and 95% humidity. Experiments were carried out when the cells reached approximately 70% confluence.

### 2.2 Cell treatment

C2C12 cells (10^5) were plated in 25 cm^2^ culture flasks and supplemented with 5 mL of culture medium. The *in vitro* shockwave (SW) treatment was performed as described by Holfeld et al. (2014). In detail, when the cells reached 70% of confluence, SW was applied via a water bath specifically designed to allow propagation of the shock waves after passing through the cell culture, as it has been suggested that waves that are not propagated will get reflected in the culture medium and will disrupt the upcoming waves as well as the cell monolayer. Therefore, the flask was placed in front of the applicator at a distance of 5 cm from the probe to the cell layer inside the water container in direct contact with pre-warmed (37°C) water. The SW treatment was applied using a shock wave generator DUOLITH® (Storz Medical AG., Tägerwilen, Switzerland) and stimulation parameters were chosen according to preliminary experiments, in order to maximize the effect of the SW, while concurrently minimizing possible negative effects. In detail, pulse repetition frequency and Energy Flux Density (EFD) were kept constant at 4 Hz and 0.1 mJ/mm^2,^ respectively, whereas the number of pulses varied from 500, 1000, and 1500 impulses. At the end of these pilot studies, the following parameters were selected: 500 impulses, EFD 0.1 mJ/mm^2^, at 4 Hz delivered in a single SW session. All the control groups were maintained in the same culture conditions without previous SW exposure. Cell treatments were performed in triplicates. Both treated and untreated cells were incubated at 37°C and subjected to biochemical and morphological analyses at various time points ranging from 24 h to 7 days following SW application.

### 2.3 Cell viability assay

Cell viability after SW stimulation was assessed by the reduction of the 2-(2-methoxy-4-nitrophenyl)-3-(4-nitrophenyl)-5-(2,4-disulfophenyl)-2H tetrazolium monosodium salt (WST-8) to a yellow-colored water-soluble formazan dye as an index of the activity of dehydrogenases in cells (Sigma Aldrich, Merk Life Science, Milan, Italy). Briefly, SW-stimulated and unstimulated C2C12 cells were plated into 96-well plates at a density of 3 × 10^4^ cells/well in a growth medium. Then, 10 μL of WST-8 solution was added to each well of the plate and incubated for 4 h at 37°C and 5% CO_2_. The absorbance at 450 nm was measured using a microplate reader (HiPo MPP-96, Biosan, Riga, Latvia). Each experimental point was performed in quintuplicate for three different experiments. Data were expressed as the mean ± S.E.M.

### 2.4 Sample preparation for transmission electron microscopy (TEM) analysis

Sample preparation protocols and analysis methods were adapted from previous studies (Fulceri et al., 2021). Briefly, at each time point (24 h, 72 h, 7d), the culture medium was removed, and a fixing solution (2.0% paraformaldehyde and 0.1% glutaraldehyde in 0.1 M Phosphate Buffered Saline (PBS), pH 7.4) was added to the cell culture and incubated for 90 min at 4°C. After fixation, the cells were gently scraped from the flask, collected into vials, and centrifuged at 10,000 for 10 min each. Subsequently, the resulting pellet was treated with 1% osmium tetroxide (OsO_4_) for 2 h at 4°C for the post-fixation process. Graded concentrations of ethyl alcohol were used to dehydrate the cell pellet. Next, propylene oxide solution was added for 15 min to enhance compatibility between the sample and the epoxy resin. The pellet was embedded overnight in a solution consisting of 50% epon-araldite and 50% propylene oxide, followed by embedding in pure epon-araldite resin (Sigma-Aldrich, St. Louis, MO, United States) at 60°C for 72 h. Ultrathin sections (90 nm) were cut at ultra-microtome (Leica Microsystems, Wetzlar, Germany) and collected on 200-mesh copper or nickel grids. Then, both plain TEM and post-embedding immunocytochemistry were carried out. Finally, grids were treated with lead citrate and uranyl acetate for dyeing. A Jeol JEM SX100 electron microscope (Jeol, Tokyo, Japan) was utilized for image analysis.

### 2.5 Morphometric analyses at TEM

Ultrastructural morphometric analysis was performed directly on the electron microscope using grids containing non-serial ultrathin sections at a magnification of ×5,000. Each grid (n = 10 for each experimental group) was scanned entirely, starting from one end to detect multinucleated cells (syncytia). Cells with at least two nuclei, exhibiting complete fusion of the plasma membranes, were identified and counted as multinucleated cells. The result was expressed as the percentage of multinucleated cells to the total number of cells counted in each grid.

### 2.6 Post-embedding immunogold labeling on ultrathin sections

Ultrathin sections collected on nickel grids were processed for protein detection within different subcellular compartments, namely, the nucleus and cytoplasm. The following primary antibodies were used: MyoD (Catalog # MA5-47019, Invitrogen, Waltham, MA, United States), MyoG (Catalog # MA5-11486, Invitrogen) (Supplementary Table S1). Ultrathin sections were first deosmicated using sodium metaperiodate (NaIO_4_). After this, they were washed with PBS (pH 7.2) and incubated for 20 min in a blocking solution composed of 10% goat serum and 0.2% saponin in PBS. Following the blocking step, the sections were incubated overnight at 4°C with a primary antibody solution, which included either rabbit anti-MyoD (dilution 1:10) or mouse anti-MyoG (dilution 1:20) (Supplementary Table S1)., both prepared with 1% goat serum and 0.2% saponin in PBS. After washing with PBS, the grids were incubated for 1 h with gold-conjugated secondary antibodies (1:20, gold particles mean diameter 20 nm, BB International, Cardiff, United Kingdom). After rinsing in PBS, grids were incubated with 1% glutaraldehyde for 3 min. Sections were stained with uranyl acetate followed by lead citrate and examined under the electron microscope.

### 2.7 Quantitative analysis of immunogold subcellular localization

Subcellular distribution of both MyoD and MyoG immunoreactivity was quantified by counting the gold particles within the nucleus and the cytoplasm at a magnification of ×8,000 on grids containing non-serial ultrathin sections. Several grids were analyzed to obtain a total of 50 cells for each experimental group. All counts were performed manually by an investigator in a blind-folded fashion. The distribution of gold particles is expressed as the percentage of particles within the nucleus or cytoplasm relative to the total number of particles in both cellular compartments (nucleus + cytoplasm).

### 2.8 Histomorphometric analysis of cell size on stained semithin sections

Semithin sections (1–2 µm) of Epon-embedded cells were cut at an Ultra Microtome (Porter Blum MT-1), collected with a metal loop, and transferred to a drop of purified water on a glass slide. After being wholly dried on a hot plate, sections were covered with a drop of Toluidine blue stain solution (with the heat source still on) for 1–2 min and then rinsed gently with distilled water to remove the excess stain. Once air-dried, the slides were coverslipped with mounting medium and observed under a Nikon Eclipse 80i light microscope (Nikon, Tokyo, Japan). Cell length and width measurements were performed in 100 cells/group with ImageJ software 1.52v.

### 2.9 Western blot

Lysates of C2C12 control and SW-treated cells were obtained using a lysis buffer (50 mM Tris, 2 mM EDTA, 100 mM NaCl, 1% NP40) supplemented with a protease inhibitor cocktail 1× (S8830, Sigma Aldrich) at 4°C. Samples were then homogenized by using three 10s pulses applied with a probe-tip ultrasonicator (SONOPLUS mini20, Bandelin) (set at 4.0 W corresponding to 14,400 J) in an ice bath. Lysates were centrifuged at 15,000 rpm for 20 min at 4°C, and protein concentration in supernatants was determined by the bicinchoninic acid assay (BCA) (Pierce™, Thermo Fisher Scientific) microplate method. Proteins (30 µg/lane) were separated under reducing conditions on a 4%–20% polyacrylamide gel (BioRad, Hercules, CA, United States) and electroblotted onto a nitrocellulose membrane using the Trans Turbo Blot system (BioRad). Aspecific binding sites were blocked by incubating membranes in EveryBlot buffer (BioRad) for 5 min, and after three washings in Tris Buffered Saline solution containing 0.1% Tween 20 (T-TBS) of 5 min each, the membranes were incubated overnight at 4°C with the following antibodies diluted in T-TBS containing 5% dry fat milk: 1) Rabbit anti-MyoD 1:1000; 2) Mouse anti-Myogenin 1:500; 3) Rabbit anti-GAPDH (G9545, Sigma Aldrich) as protein loading control (Supplementary Table S1). Anti-Rabbit- or anti-Mouse-HRP-conjugated secondary antibodies (BioRad), diluted 1:5000 in T-TBS containing 5% of dry fat milk, were used, and the immunocomplexes were detected by chemiluminescence (ECL clarity, BioRad) by using Chemi-Doc XR (BioRad). The intensity of the bands was measured using the Image Lab Software (BioRad) on n = 3 different series of samples. All reactions were performed at room temperature unless otherwise specified.

### 2.10 Statistical analysis

All data are presented as the mean or mean percentage ±standard error of the mean (S.E.M.). Statistical analysis was performed with StatView software (version number: 5.0.1). Comparisons between groups were analyzed using one-way analysis of variance (ANOVA) followed by Scheffè’s *post hoc* analysis, and for Western blot analysis, the Tukey HSD *post hoc* test was employed. The null hypothesis (H_0_) was rejected for p < 0.05.

## 3 Results

### 3.1 Myoblast response to SW application of different parameters

**FIGURE 1:**
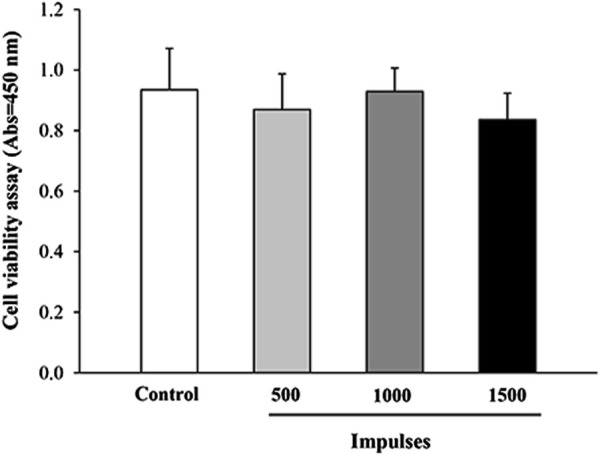
Cell viability assessment by WST-8 assay. The viability of C2C12 cells was evaluated 24 h after exposure to different SW pulses using the WST-8 assay. Data are presented as mean ± SEM from three independent experiments.

Preliminary studies were conducted to assess the impact of shockwave (SW) application at varying energy levels on cell viability, using the WST-8 assay. As shown in Figure 1, no significant differences in cell viability were observed between the treatment groups 24 h after SW application. These results were further corroborated by a trypan blue exclusion assay, which similarly showed no significant differences in cell viability between groups (Supplementary Figure S1). Although no viability differences were observed, high-energy treatments could potentially induce subtle stress responses in the cells. This is in line with previous studies (Hansen et al., 2017; Mattyasovszky et al., 2018), which showed that the application of low-dose SW (below 1000 pulses) was well tolerated by myoblast cell culture as it did not exert harmful effects on the cells. Therefore, the following SW parameters were selected as the regimen of use for the subsequent biochemical and ultrastructural investigations: 500 impulses, EFD 0.1 mJ/mm^2^, at 4 Hz delivered in a single SW session.

### 3.2 SW application induces morphological changes in myoblast

**FIGURE 2:**
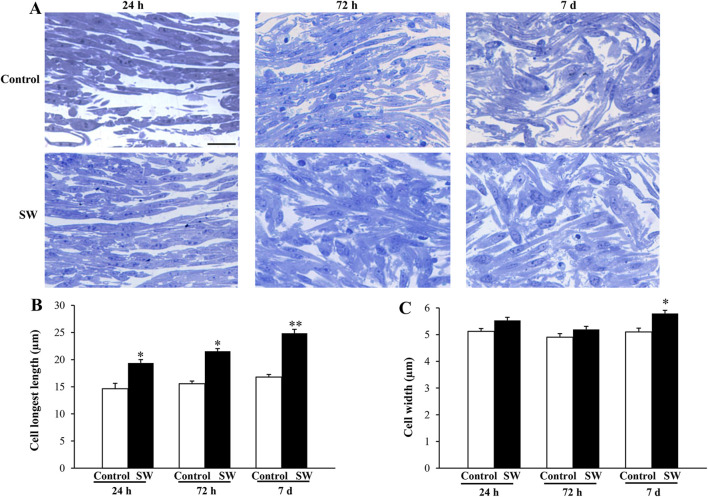
Morphological changes induced by SW treatment. **(A)** Representative picture of toluidine-stained semithin sections of control cells and after SW exposure at different time points (magnification ×20). Morphometric changes in the cell’s length **(B)** and width **(C)** following SW exposure are reported in the graphs, showing that SW-treated cells become more elongated and thicker than controls. Cell length and width measurements were performed in 100 cells/group. Scale bar = 20 μm **(B)** *p < 0.05 vs. control (SW 24h, 95% CI: 14.62 to 16.51; *p* < 0.0001; SW 72h, 95% CI: 18.15 to 20.60; *p* < 0.0001); **p < 0.05 vs. other groups (SW 7d, 95% CI: 23.44 to 26.27; *p* < 0.0001); **(C)** *p < 0.05 vs. controls and SW 72h (SW 7d, 95% CI: 5.55 to 6.03; *p* = 0.0081).

Previous studies have shown that, during the differentiation process from myoblast into multinucleate myotubes, C2C12 cells undergo remodeling and progressively change their morphology to adopt a more elongated and fusiform shape (Burattini et al., 2004; Casadei et al., 2009; Niioka et al., 2018). Indeed, these profound changes in cell shape and cellular elongation serve as early indicators of myoblast differentiation that precede cell fusion. Therefore, to evaluate the effects of SW on cell morphology, toluidine-blue stained semithin sections were observed at light microscopy. As reported in Figure 2, undifferentiated control cells were recognizable as flat, rounded, or fusiform, and no changes in cell morphology were observed over time (Figure 2A, upper panels). In contrast, the morphological changes were evident in SW-treated cells, which exhibited noticeable differences compared to the controls, becoming more pronounced over time (Figure 2A, lower panels). Indeed, morphometric analysis at light microscopy showed a significant increase in the cell size of treated cells compared to controls, showing a more elongated and thicker appearance. This is even more evident in SW-treated cells at 7 days post-treatment, where the most noticeable increase in cell size occurred. Morphometric analysis of the SW-treated cells was accomplished by measuring the longest length and width of individual cells as reported in Figure 2B. Compared to controls, there was a significant increase in the average cell length following SW treatment at each time point (SW 24 h, 95% CI: 14.62 to 16.51*; p* < 0.0001; SW 72h, 95% CI: 18.15 to 20.60*; p* < 0.0001; SW 7d, 95% CI: 23.44 to 26.27*; p* < 0.0001). Similarly, there was a tendency to increase in cell width, which was significant at 7 days post-treatment (95% CI: 5.55 to 6.03*; p* = 0.0081).

### 3.3 SW application induces ultrastructural changes in C2C12 myoblasts indicative of impending cell fusion

**FIGURE 3:**
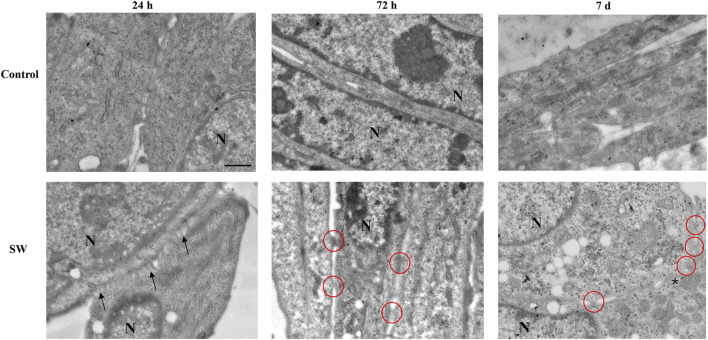
Ultrastructural morphology of C2C12 cells after treatment with SW. The representative micrographs illustrate C2C12 cells from the control culture (upper panels) compared to those after SW treatment (lower panels) (magnification ×5,000). In the control group, the cells are in close proximity with well-defined and distinct membranes. Conversely, C2C12-treated cells progressively feature ultrastructural peculiarities that precede the myoblast fusion process. In the early stage of differentiation (24 h after SW), the appearance of membrane branches (arrows) characterizes the cell surface of apposed cells. At 72 h post-SW treatment, following the establishment of adhesive contact, the plasma membranes of adjacent C2C12 cells are drawn into close proximity. Electron-dense plaques appear along the contacting membranes, highlighted by red circles, representing sites of membrane fusion activity and indicative of cells in the process of forming a syncytium. At a later time (7d after SW), an increased number of dense plaques and the fusion of focal membrane areas (asterisk) are observed. N = nucleus; Scale bar = 1 μm.

The morphological analyses were accompanied by ultrastructural observations, which provided insights into some peculiarities of SW-treated cells during their commitment toward differentiation. As shown in Figure 3, TEM analysis revealed no significant ultrastructural changes in control cells at different time points (Figure 3, upper panels). Moreover, though cells appear in close proximity with reduced intercellular space, no fusion points along cell membranes were detected at any time. In contrast, SW-treated cells exhibited clear ultrastructural features of an ongoing myoblast fusion that precedes the formation of multinucleated syncitia. Notably, 24 h after SW treatment, numerous thin, protrusive structures emanating from apposed myoblast membranes were observed (Figure 3, lower panels). These “finger-like” projections bring membranes in closer proximity, facilitate their alignment, and initiate cell-cell contacts, marking the earliest step in the formation of fusion sites. As fusion progresses (72 h), the plasma membrane of opposing aligned myoblasts begins to merge, forming localized cell-cell contacts and membrane-associated electron-dense plaques. Over time (7d), multiple fusion sites appear, which then expand laterally, leading to membrane breakdown and the establishment of full cytoplasmic continuity between adjacent cells.

### 3.4 SW treatment increases the percentage of multinucleated C2C12 cells

**FIGURE 4:**
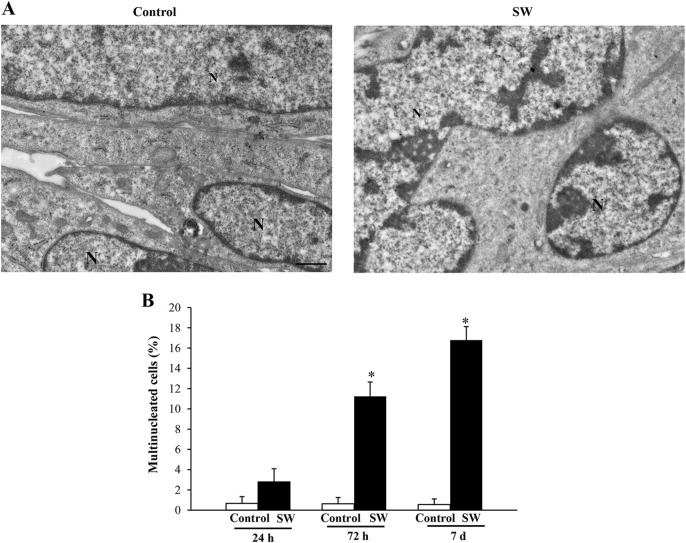
SW treatment induces the formation of multinucleated C2C12 cells. Representative micrographs of C2C12 cells in control and SW-treated conditions (magnification ×5,000). **(A)** In the control group, three adjacent C2C12 cells are distinctly visible, each exhibiting well-defined plasma membranes that delineate individual cell boundaries. In contrast, the SW-treated group shows the formation of a syncytium, characterized by the fusion of plasma membranes and cytoplasmic continuity between fusing cells, indicating successful myogenic differentiation and cell fusion. **(B)** Graph reports the percentage of multinucleated cells found in the control and after SW (n = 10 grid/group). N = nucleus; Scale bar = 1 μm; *p < 0.05 vs. other groups (SW 72h, 95% CI: 8.47 to 14.01, *p* < 0.0001; SW 7d, 95% CI: 14.20 to 19.39; *p* < 0.0001).

The SW-induced myoblast differentiation was further confirmed by the ultrastructural evidence of the transition from C2C12 mononucleated cells to early syncytial structures containing a small number of nuclei. Indeed, quantification of myogenic potency expressed as the number of syncytia characterized by the presence of at least two nuclei demonstrated substantial differences between controls and treated cells at different time points (Figure 4A). As shown in Figure 4B, the percentage of multinucleated cells found in the control group was below 1% of the total cell number over the entire experimental period. In contrast, exposure of C2C12 myoblasts to SW was shown to increase the number of multinucleated cells already at 72 h post-SW application (95% CI: 8.47 to 14.01*; p* < 0.0001), reaching approximately 11%. This increase was even more pronounced at the later time point, where the percentage of multinucleated cells in the experimental cultures was over 16% (95% CI: 14.20 to 19.39; *p* < 0.0001), greatly exceeding those observed in controls.

### 3.5 SW treatment does not alter the expression of myogenic differentiation markers

**FIGURE 5:**
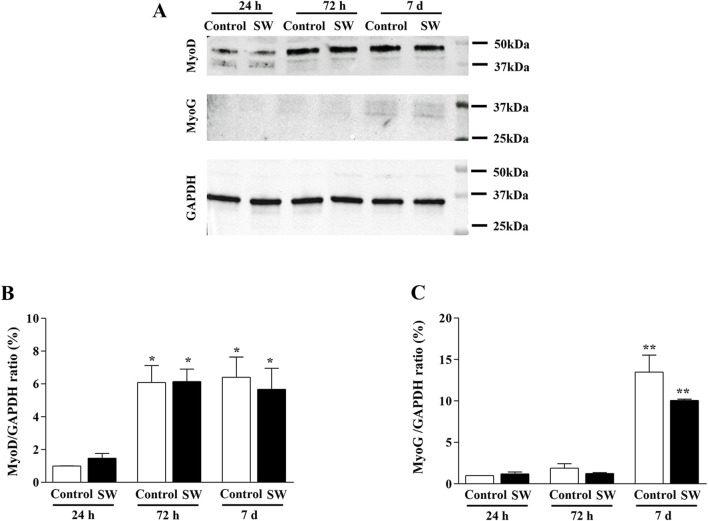
Time-series comparison of the protein expression of MyoD and MyoG after SW treatment. **(A)** Representative western blots of MyoD, MyoG, and GAPDH. **(B,C)** Densitometric analysis of Western blot was normalized to GAPDH used as internal control. Results are reported as mean ± S.E.M. of n = 3 experiments. *p < 0,05 vs. Control 24 h and SW 24 h (SW 72h, 95% CI: 4.66 to 7.64; *p* = 0.018; SW 7d, 95% CI: 3.16 to 8.18; *p* = 0.0018), **p < 0.001 vs. control 24h, control 72h, SW 24h, and SW 72 h (SW 7d, 95% CI: 9.75 to 10.37; *p* < 0.0001).

Given our observation of morphological and ultrastructural changes, we investigated whether SW modulated the expression of myogenic markers (MyoD and MyoG) at different time points (24 h, 72 h, and 7d after SW application) (Figure 5A). This approach, in turn, allowed us to assess the baseline expression of these markers in undifferentiated myoblasts and track their changes during the early differentiation stages toward the formation of multinucleated syncytia. However, Western blot analysis revealed no significant differences in total MyoD and MyoG protein expression between SW-treated cells and controls over time (Figures 5B,C), as the treatment did not alter the expression of both markers. Notably, similar to controls, C2C12-treated cells exhibited the typical pattern of expression and sequential roles of myogenic markers, where MyoD levels increased at early time points (SW 72h, 95% CI: 4.66 to 7.64*; p* = 0.018; SW 7d, 95% CI: 3.16 to 8.18*; p* = 0.0018), reflecting its role in cells committed to the myogenic lineage (Figure 5B), while MyoG expression, which regulates the later stages of myogenic differentiation, showed a delayed increase, peaking only at 7d post-treatment (SW 7d, 95% CI: 9.75 to 10.37*; p* < 0.0001) (Figure 5C).

### 3.6 SW promotes nuclear compartmentalization of MyoD and MyoG proteins in C2C12 cells

**FIGURE 6:**
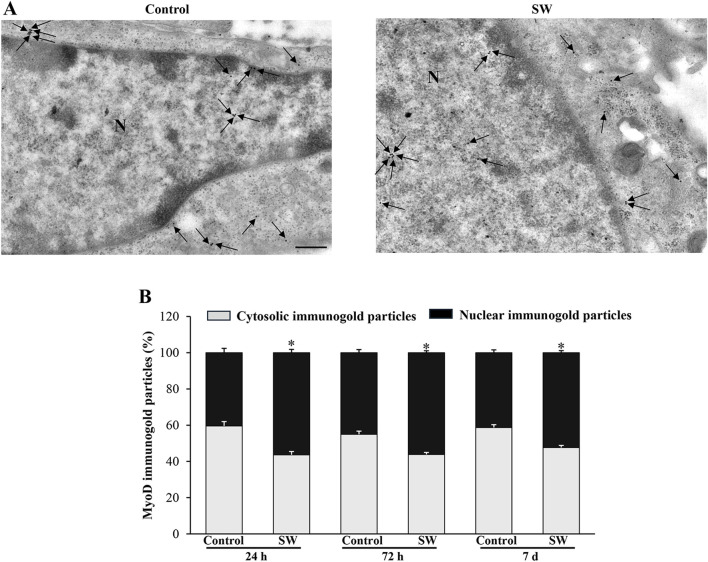
SW promotes nuclear compartmentalization of MyoD protein in C2C12 cells. **(A)** Representative TEM micrographs showing MyoD immunogold particles within C2C12 cells from controls and after SW treatment (magnification ×8,000). Black arrows point to immunogold particles 20 nm Ø. **(B)** Graph reports the percentage of MyoD particles within the nucleus and the cytosol (n = 50 cells/group). N = nucleus; Scale bar = 200 nm; *p < 0.05 vs. control (SW 24h, 95% CI: 52.76 to 59.95; p < 0.0001; SW 72h, 95% CI: 53.98 to 58.30; p = 0.0008; SW 7d, 95% CI: 50.04 to 54.60; p = 0.0010).

**FIGURE 7:**
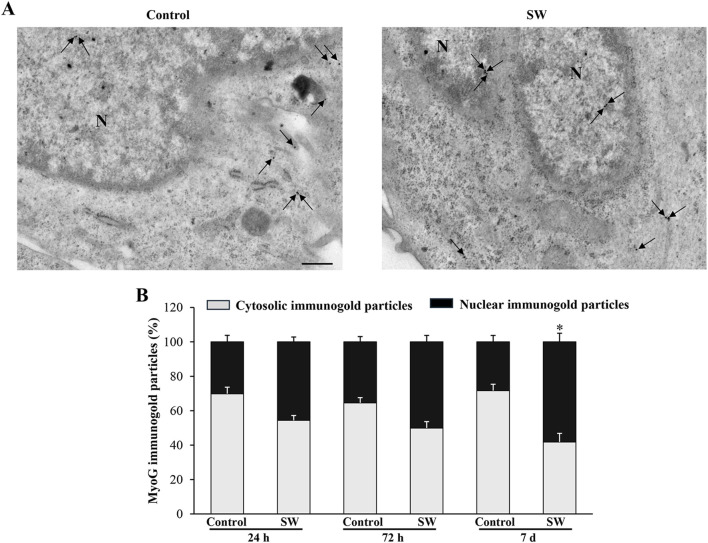
SW promotes nuclear compartmentalization of MyoG protein in C2C12 cells. **(A)** Representative TEM micrographs showing MyoG immunogold particles within C2C12 cells from controls and after SW treatment (magnification ×8,000). Black arrows point to immunogold particles 20 nm Ø. **(B)** Graph reports the percentage of MyoG particles within the nucleus and the cytosol (n = 50 cells/group). N = nucleus; Scale bar = 200 nm; *p < 0.05 vs. control 7d (SW 7d, 95% CI: 48.35 to 68.04; p < 0.0001).

Since the morphological and ultrastructural alterations induced by SW did not align with the immunoblotting results regarding the transcriptional activity of the myogenic differentiation markers, we examined the subcellular distribution of MyoD and MyoG immunoreactivity using immunogold-based electron microscopy. This approach was crucial as the transcriptional activity of MyoD and MyoG strictly depends on their translocation to the nucleus. Remarkably, quantitative assessment of immunogold labeling revealed that the nuclear localization of both MyoD and MyoG gold particles was significantly improved following SW treatment. Specifically, the percentage of immunogold particles (indicating protein presence) located within the nuclear compartment was greater in treated cells compared to controls (Figures 6, 7). In detail, in untreated controls, MyoD was mainly distributed within the cytoplasm (Figure 6A). However, after SW exposure, a noticeable increase in its nuclear compartmentalization was observed (Figures 6A,B; Supplementary Figure S2A). A similar pattern was observed for MyoG, which also showed mostly cytoplasmic localization in control cells, but exhibited a clear shift towards the nucleus following SW exposure (Figures 7A,B; Supplementary Figure S2B). Interestingly, the degree of SW-induced nuclear compartmentalization was greater for MyoD than for MyoG. As shown in the graphs of Figures 6, 7, nuclear MyoD levels were already markedly increased at 24 h post-treatment compared to controls (Figure 6B; SW 24 h, 95% CI: 52.76 to 59.95; p < 0.0001; SW 72 h, 95% CI: 53.98 to 58.30; p = 0.0008; SW 7d, 95% CI: 50.04 to 54.60; p = 0.0010), whereas a statistically significant increase in nuclear MyoG was observed only at a later time point (Figure 7B; SW 7d, 95% CI: 48.35 to 68.04; p < 0.0001). This pattern is consistent with the sequential roles of these myogenic markers during the early stages of myogenic differentiation.

## 4 Discussion

**FIGURE 8:**
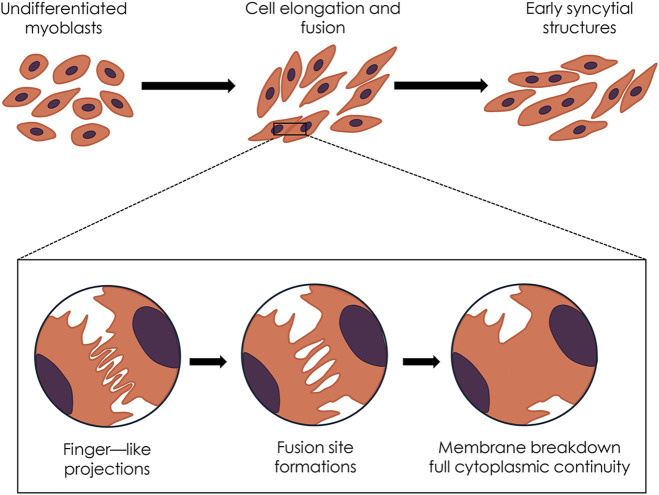
Diagram summarizing the hypothesized sequence of shockwave-induced morphological and ultrastructural events.

In the last decades, extracorporeal shockwave therapy (ESWT) has emerged as a non-invasive and safe therapeutic modality for treating several orthopedic and musculoskeletal pathologies (Moya et al., 2018). While ESWT has proven effective in promoting healing in bones, tendons, and joint pathologies, its impact on muscle tissue repair and regeneration remains less understood. Indeed, there is limited evidence of its effectiveness both in clinical and experimental settings (Astur et al., 2015; Fleckenstein et al., 2016; Zissler et al., 2017; Huang et al., 2021; Lee et al., 2023). Remarkably, some findings on animal models indicate that ESWT may promote an acceleration of muscle healing (Kisch et al., 2016; Zissler et al., 2017; Wang et al., 2024). However, the biological mechanisms underlying shockwaves (SW) have remained largely unexplored, and only a few studies on the *in vitro* effects of SW on myoblast cells are currently available (Hansen et al., 2017; Mattyasovszky et al., 2018; Wang et al., 2017). At the same time, as far as we know, a detailed ultrastructural analysis of myoblast cells exposed to SW and morphometric analysis of protein subcellular distribution following SW treatment has never been performed. In particular, in the present study, we demonstrate that SW is capable of promoting *in vitro* C2C12 myoblast fusion and differentiation, as evidenced by morphological and ultrastructural changes. Moreover, we identified that the nuclear translocation of myogenic markers MyoD and MyoG contributes to this promising effect mediated by SW (Figure 8). These findings highlight the potential of SW as a stimulus to influence the biology of myoblast cells and promote their differentiation, which could have important implications for regenerative medicine and muscle repair.

A crucial event in myogenesis is the cell-cell fusion of mononucleated myoblasts to form multinucleated syncytia (Sampath et al., 2018; Lehka and Rędowicz, 2020). This, in turn, can be divided into several key stages where myoblasts undergo remodeling, including, for instance, cell elongation, membrane apposition, fusion pore formation, and ultimately, membrane merging (Abmayr and Pavlath, 2012; Leikina et al., 2013). Remarkably, data from the present research showed that SW treatment can induce a significant increase in cell size and elongation, both of which are key events during *in vitro* myoblast differentiation and precede myoblast fusion (Ohtake et al., 2006). As shown by previous studies, upon appropriate stimuli including mechanical stimulation, C2C12 cells typically undergo a remodeling process during differentiation, transitioning towards a more fusiform and elongated morphology (Burattini et al., 2004; Casadei et al., 2009; Grossi et al., 2011; Wang et al., 2014; Espinoza-Álvarez et al., 2024). Similarly, in our study SW-treated cells displayed noticeable changes in shape, with a marked increase in cell size, as quantified through morphometric analysis. The morphological changes induced by SW exposure were significantly more pronounced compared to controls by 7d post-treatment, suggesting that SW may accelerate the differentiation process.

Ultrastructural investigations corroborate the morphological evidence of an early myogenic commitment of C2C12 cells following SW stimulation. Furthermore, the high-resolution TEM analysis provided compelling evidence that SW stimulation can trigger a sequence of membrane-associated events that culminate in the fusion of C2C12 myoblasts. Unlike untreated controls, SW-treated cells display key features of impending fusion, such as membrane protrusions and localized contact sites, which precede the formation of nascent multinucleated syncytial structures. In detail, at early time points (24 h) following SW stimulation, we could detect the occurrence of finger-like protrusions at the fusion site of C2C12 cells, which in contrast are absent in fusion-incompetent control cells. This feature allowed us to document “*en face*” the SW-induced fusion process as these structures are key intermediates of early fusion events as they facilitate the alignment of opposing membranes, a crucial prerequisite for successful cell-cell fusion. This observation resonates with findings by Le Clainche and Carlier (Le Clainche and Carlier, 2008), who described the critical role of acting remodeling and filipodia-like structures in membrane apposing during fusion, as well as more recent works that emphasize the functional role of cytoskeletal protrusion in mediating cell-cell recognition, contact stabilization, and thus efficient fusion (Randrianarison-Huetz et al., 2018; Cong et al., 2019; Lu et al., 2024a; 2024b).

As fusion progresses, the plasma membrane of opposing aligned myoblasts begins to merge, forming localized cell-cell contacts and membrane-associated electron-dense plaques. Over time, multiple fusion sites appear, which then expand laterally, leading to membrane breakdown and the establishment of full cytoplasmic continuity between adjacent cells. Furthermore, at 72 h post-treatment, we observed an increased number of discrete fusion points characterized by aligned plasma membranes and the occurrence of electron-dense plaques, indicative of nascent fusion machinery assembly. These sites later (7d post-treatment) expanded to form wider zones of membrane continuity, leading to the dissolution of intervening membranes and the formation of nascent syncytial structures. The identification of these features at TEM, especially in a sequential and time-dependent manner, provides unique ultrastructural evidence of the dynamic fusion landscape induced by this mechanical stimulation. Interestingly, while most studies on myoblast fusion focus on the biochemical and genetic modulation of fusogenic proteins, only a few reports have addressed the morphological and ultrastructural underpinnings of this process, and particularly in response to a mechanical stimulus such as shockwave. Within this frame, the results of the present study bridge this gap offering a detailed ultrastructural framework for understanding how SW exposure can prime and accelerate myogenic fusion through the induction of membrane protrusion and the establishment of localized fusion domains. These findings are in line with emerging literature suggesting that mechanical forces and membrane tension are key modulators of fusion competence in muscle cells (Kim et al., 2015; Chakraborty et al., 2022; Millay, 2022). The prolonged presence and maturation of fusion sites observed at later time points (up to 7d) suggest that SW stimulation not only can initiate early membrane contact but also sustains the fusion process, possibly through the modulation of membrane and cytoskeletal dynamics. Further studies on the possible effects of SW in the coordination of known fusogenic proteins may bring novel insights into the biophysical regulation of myoblast fusion and differentiation.

Additionally, the TEM results observed in this study align with the evidence of an increased proportion of multinucleated cells following SW exposure, which are detectable as early as 24 h post-treatment and significantly increased at later stages. This further supports the progression toward myogenic differentiation and the formation of syncytial structures. However, our data contrast with those of Hansen et al. who found no significant effect on the *in vitro* differentiation of primary isolated human myoblasts treated with low-intensity SW (Li-SW) (Hansen et al., 2017). Interestingly, when performing the *in vivo* experiment, the same authors reported a significantly increased expression of myogenic genes in the early phase of regeneration in the Li-SW-treated hind limbs after acute injury. On the other hand, Mattyasovszky et al. documented morphological changes similar to those in our study, where Li-SW was shown to enhance *in vitro* myotube formation in L6 rat myoblasts (Mattyasovszky et al., 2018). The inconsistencies between the above-reported data are not so unexpected since a significant body of literature highlights the challenges of comparing results due to the high variability in treatment protocols and type of SW devices used. This issue does not only apply to *in vivo* studies but is even more evident when comparing *in vitro* data. Indeed, the controversial data from the *in vitro* studies may be explained by the heterogeneous experimental setting including differences in cell types and above all the variable application method of the shockwaves. This variability underscores the need for the establishment of standardized *in vitro* treatment protocols to improve the reproducibility and comparability of future studies.

Despite the observed morphological and ultrastructural changes, our study found no significant alteration in the total levels of the myogenic markers between SW-treated cells and controls. This finding is noteworthy, as MyoD and MyoG are pivotal in regulating myoblast differentiation and fusion (Bentzinger et al., 2012). MyoD is known to initiate the myogenic program, while MyoG regulates the later stages of differentiation (Hernández-Hernández et al., 2017). Previous studies have reported variable effects of mechanical stimuli on the expression of these markers, with some studies showing increased MyoD and MyoG expression in response to external stimuli (Grossi et al., 2007; Abe et al., 2009; Chan et al., 2023), while others have observed no significant changes (Kook et al., 2008; Kuang et al., 2009; Wang et al., 2020). Our results suggest that while SW treatment does not significantly alter the overall levels of the myogenic markers, it may still facilitate myogenesis by promoting the transition from mononucleated to multinucleated cells. This is likely due to the mechanical cues provided by SW, which may activate signaling pathways involved in myoblast fusion without altering MyoD and MyoG levels.

Indeed, one of the most striking findings in the present study was the enhanced nuclear localization of MyoD and MyoG following SW treatment. Nuclear translocation is critical for both of these transcription factors to exert their function (Bentzinger et al., 2012). Previous studies by Ferri et al. (2009) showed that myogenic regulatory factors (MRFs) nucleo-cytoplasmic trafficking is involved in muscle differentiation, suggesting that, besides their expression levels, MRF subcellular localization, related to their functional activity, also plays a key role as a regulatory step in transcriptional control mechanisms. Consistent with their transcriptional activity, immunogold electron microscopy data revealed that SW may have the potential to influence the biology of myoblast cells by modulating MRF subcellular localization. In detail, we observed a significant increase in the nuclear localization of both markers in SW-treated cells, with MyoD showing a more prominent shift compared to MyoG. Remarkably, the increase in nuclear MyoD localization observed in the present study is similar to that reported by Chandorkar et al. (2022), who demonstrated that mechanical stimulation enhances the nuclear accumulation of this transcription factor in muscle cells. In contrast, MyoG nuclear shuttling was less pronounced and occurred at later time points, which again aligns with the sequential roles of these myogenic markers.

Thus, it can be speculated that SW was not capable of increasing the expression of myogenic markers, but rather acted by favoring the nuclear localization of these transcription factors to drive the myogenic program. Again, the sequential pattern of MRFs’ nuclear subcellular localization suggests that SW treatment can accelerate the differentiation process by facilitating the early stages of myogenesis, leading to the rapid formation of multinucleated cells.

**FIGURE 9:**
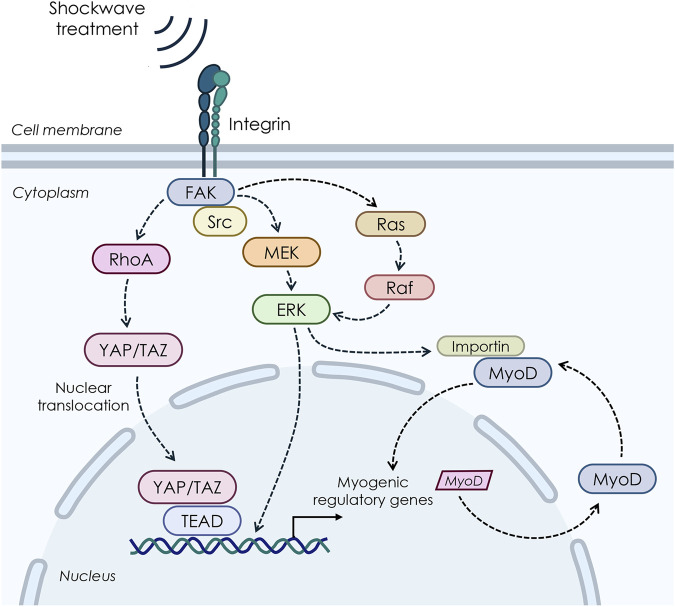
Possible pathways involved in the biological response to shockwave stimulations on myoblast cells.

At present, there is still limited evidence specifically addressing the mechanobiology of shockwave therapy concerning upstream signaling pathways and nucleocytoplasmic shuttling. Although mechanistic investigations were beyond the scope of the present study, insights can be drawn from related research on mechanical stimuli and related cellular responses, which allow us to hypothesize potential upstream pathways, including YAP/TAZ, MAPK, and integrin-mediated signaling pathways, and which warrant further exploration in future studies (Figure 9).

Within this frame, our ultrastructural data are in line with previous studies showing that mechanical cues can enhance the nuclear import of specific proteins and transcription factors, thereby influencing multiple essential pathways in the cell (Tothova et al., 2006; Montel et al., 2019; Kong et al., 2021; García-García et al., 2022). For instance, research on vascular smooth cells has shown that nuclear protein import was significantly stimulated in stretched cells when compared with control cells through a mitogen-activated protein kinase (MAPK)-mediated mechanism, facilitating cell growth (Richard et al., 2007). Of note, MAPK/extracellular signal-regulated kinase (ERK) signaling pathway is known to be involved in mechanically induced signaling in various cell types, including skeletal muscle, where MAPK/ERK pathway has been reported to mediate the transmission of extracellular signals to the nucleus in response to contraction and passive stretch (Martineau and Gardiner, 1985). At the same time, *in vitro* studies on smooth muscle cells have demonstrated that the MAPK pathway is involved in the stretch-induced stimulation of nuclear protein import and increased nuclear pore expression (Richard et al., 2007). In line with this, a few studies have suggested that shockwaves produce mechanical stimulation through pressure change, thus initiating a serial cellular function by triggering various intracellular signaling events that involve MAPK signaling (Weihs et al., 2014; Catalano et al., 2017; Shen et al., 2021). For instance, research on porcine articular chondrocytes revealed that the transient production of ROS induced by SW can activate the MAPK/Nfr2 signaling, thus leading to the phosphorylation of ERK1/2 and p38, Nrf2 nuclear translocation, and subsequent activation of the expression of genes encoding various antioxidant enzymes (Shen et al., 2021). By contrast, pretreatment of chondrocytes with the specific inhibitors of MEK1/2 and p38, respectively, mitigated the SW-induced nuclear translocation of Nrf2 and ECM synthesis. Again, Nrf2 inhibition by both small hairpin RNA knockdown and brusatol reduced the SW-enhanced ECM synthesis (Shen et al., 2021).

Moreover, a number of studies have demonstrated that low-intensity SW interacts with integrin and activates FAK by phosphorylation mediated by integrin α5 and β1, thereby triggering a series of cellular signaling and related biological changes, which are known to be relevant for myoblast differentiation by regulating promyogenic factors (Han et al., 2011). Again, the Wnt/β-catenin was shown to be an additional mediator in the integrin-FAK signaling pathway in response to SW in a cell culture model of osteoblasts (Xu et al., 2012).

Finally, another potential network that may link shockwave-mediated mechanosensing to gene transcription is the Hippo signaling pathway, whose central components are Yes-associated protein (YAP) and the homologous transcriptional coactivator with PDZ-binding motif (TAZ), which exhibits a strong structural similarity and genetic redundancy (Low et al., 2014). In particular, YAP/TAZ plays an important role as a mechanosensitive effector that induces cell-specific transcriptional programs as an adaptation to dynamic mechanical cues (Dupont et al., 2011; Aragona et al., 2013; Li H. X. et al., 2021; Pan et al., 2024). In line with this, previous mechanistic *in vitro* studies on subchondral bone stem/progenitor cells (SCB-SPCs) have demonstrated that YAP undergoes activation and nuclear translocation after radial SW stimulation, promoting self-renewal of SCB-SPCs (Zhao et al., 2021). Furthermore, a recent study by Li Y. et al. (2021) demonstrated that low-intensity ESWT upregulates the expression level and nuclear translocation of YAP, which acts as an integrated transcriptional complex for related gene expression modification, thereby promoting the activation of rat Schwann cells (SCs). The following SCs activation process promotes nerve axon regeneration, which might lead to functional recovery after peripheral nerve injury. Contrariwise, *in vitro* knockdown of TAZ by small interfering RNA (siRNA) significantly attenuated this process.

Although the present data increase our understanding of SW-induced myoblast differentiation, the picture is still far from clear, and further mechanistic studies are needed to fully understand the mechanisms induced by SW.

## 5 Conclusion

The present study brings new insights into the SW-induced regulation of myoblast fusion and early myogenic differentiation, while raising intriguing questions regarding the contribution of mechanical stimulation to the modulation of myogenesis. However, some limitations should be noted. First, we used an immortalized myoblast cell line, namely, the C2C12 cell line. Although this cell line is a well-established and widely used model for studying skeletal muscle cell biology, it does have some limitations, particularly regarding the translatability of findings to human muscle physiology. Therefore, experiments using also primary murine or human myoblast cells should be considered for future investigations to further extend our findings. Another limitation is the therapy schedule. In the present research, cells were stimulated only once with SW, whereas in clinical practice, multiple weekly sessions of shockwave treatment are generally recommended for the patients. Therefore, in the follow-up studies, additional frequencies, number of impulses, and/or applications should be investigated, in order to better translate the *in vivo* schedule treatment to our *in vitro* model. Furthermore, research is required to determine the upstream signaling cascade of SW-induced myoblast commitment toward early myogenic differentiation.

## Data Availability

The raw data supporting the conclusions of this article will be made available by the authors, without undue reservation.
