# Supplementary material for: Ultrastructural insights into early myoblast differentiation induced by shockwave stimulation
Source: Front Physiol. 2025 Jul 23;16:1636931. doi: 10.3389/fphys.2025.1636931 (PMC12325262; doi:10.3389/fphys.2025.1636931)
Supplement: Supplementary file 1 [file Supplementaryfile1.docx]

**Supplementary Figure 1. Measurement of cell viability.**

Cell viability was determined using the LUNA-III™ Automated Cell Counter (Logos Biosystems). Briefly, 25 μL of sample was mixed with 25 μL of 0.4% trypan blue by gently pipetting, and then 10 μL of the mix was loaded into each chamber of LUNA^TM^ cell counting Slides. Counts were performed in quadruplicate. Cell viability was normalized to the control. Data are presented as the mean ± standard error of the mean (SEM). Comparisons between groups were performed with one-way analysis of variance (ANOVA) followed by Scheffè’s post-hoc analysis. The null hypothesis (H0) was rejected for **p*<0.05.
