# Supplementary material for: Ultrastructural insights into early myoblast differentiation induced by shockwave stimulation
Source: Front Physiol. 2025 Jul 23;16:1636931. doi: 10.3389/fphys.2025.1636931 (PMC12325262; doi:10.3389/fphys.2025.1636931)
Supplement: Supplementary file 2 [file Table1.docx]

**Supplementary Table 1. Comprehensive list of antibodies used in the present study.**
