# Supplementary material for: Ultrastructural insights into early myoblast differentiation induced by shockwave stimulation
Source: Front Physiol. 2025 Jul 23;16:1636931. doi: 10.3389/fphys.2025.1636931 (PMC12325262; doi:10.3389/fphys.2025.1636931)
Supplement: Supplementary file 3 [file Table2.docx]

**Supplementary Figure 2. SW promotes nuclear compartmentalization of MyoD and MyoG proteins in C2C12 cells.**


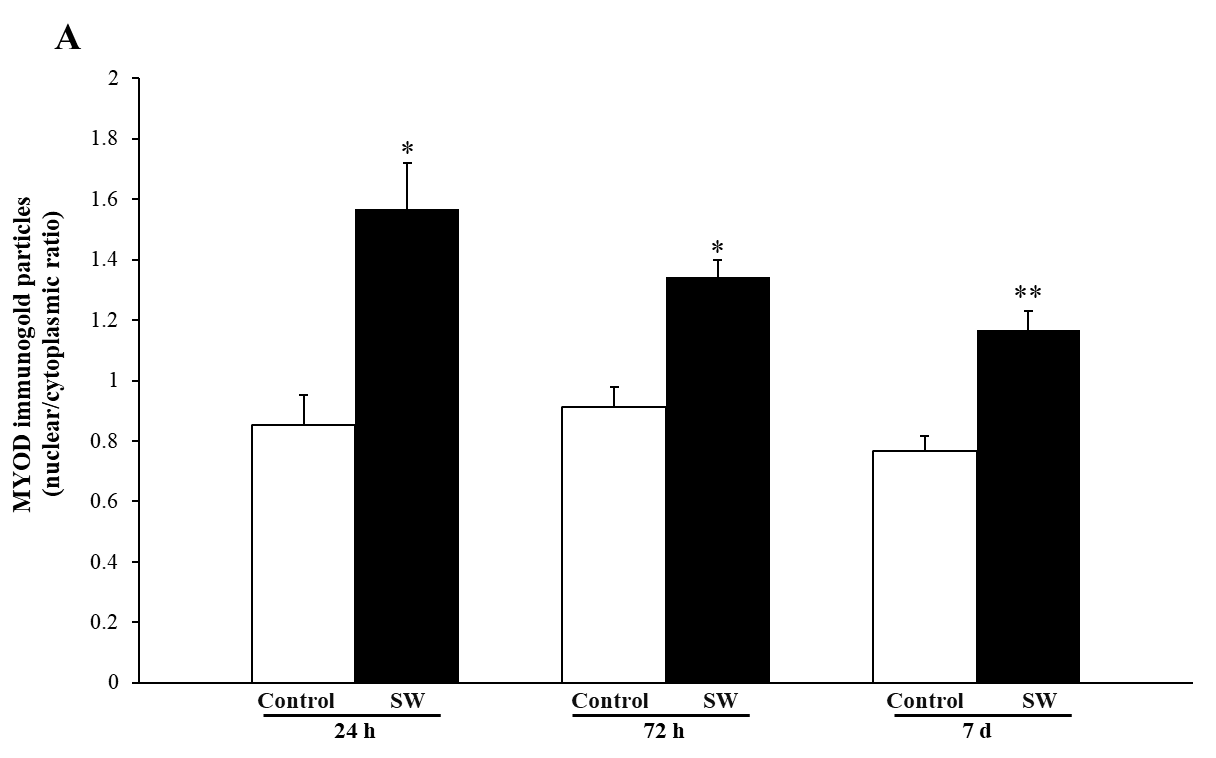


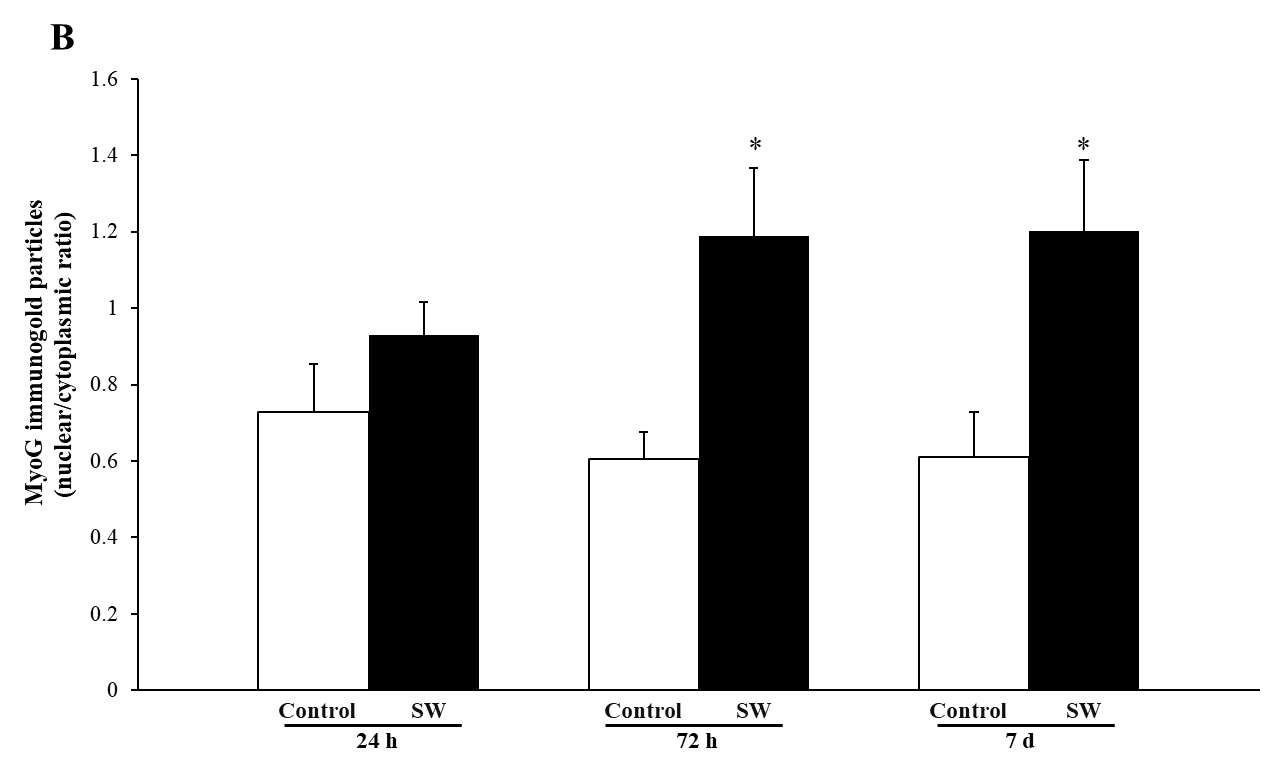


The nuclear/cytoplasmic ratio of each protein (**A**, MyoD; **B**, MyoG) was calculated as the ratio of the amount of immunogold particles in the nucleus divided by the amount of immunogold particles in the cytoplasm. Counts were performed in 50 cells. Data are presented as the mean ± standard error of the mean (SEM). Comparisons between groups were performed with one-way analysis of variance (ANOVA) followed by Fisher post-hoc analysis. The null hypothesis (H0) was rejected for **p*<0.05 vs control; ***p*<0.05 vs control and SW 24h.
